# Memoirs of the Medico-Chirurgical Society of Bologna

**Published:** 1838-01

**Authors:** 


### Art. XII.

*Memorie della Società Medico-Chirurgica di Bologna.* Vol. I. Fasc. 1, 2. —*Bologna,* 1835, 1836.

*Memoirs of the Medico-Chirurgical Society of Bologna.* Vol. I. Fasciculus 1 and 2.—*Bologna,* 1835-6. 8vo. pp. 223; with Plates.

This Society has published, for the last nine years, the *Bullettino delle Scienze Mediche,* a monthly Journal, consisting of extracts from foreign Medical Journals, and concise Reports of the proceedings of domestic and foreign Societies; but it is only since the reestablishment of its meetings, that had been suspended from 1831 to 1834, in consequence of the political events of that period, that it has published its own Transactions in a separate form. The first fasciculus was printed in 1835, the second in 1836, and each contains five articles; which, although rather deficient in originality and importance, display a pretty accurate acquaintance with the labours of foreigners in the same departments. Indeed, we have frequently had occasion to remark, that the Italian physicians are much better read in English medical literature than our nearer neighbours, the French, whose vanity, or ignorance of what is going forward in other countries, causes their honesty to be occasionally called in question when asserting their claims to new discoveries. Although the number of papers selected for publication in the Transactions is small, yet the quantity of writers among our Bolognese brethren is far from being so; for eight out of the twelve memorials read during the session 1836 to the Academy of Sciences were upon strictly medical subjects; some of which we shall have occasion to notice among our Selections.

The first paper is by Dr. Breventani, upon the auscultatory Signs of Pregnancy, as observed by him in forty women supposed to be pregnant, who all, except one not pregnant, exhibited not only the placental murmur, synchronous with the maternal pulse, but also the double fœtal sound, of the existence of which there could be no doubt, from its similarity to the sounds of the heart in the new-born infant. The placental murmur was never heard before the third month of pregnancy. At first it was perceptible in one spot at the lower part of the hypogastric region; and, as the uterus increased in bulk, it became more marked on one side, usually the right, and over the uterine enlargement; always preserving, however, its relative position with regard to the uterus. At first the sound is continuous, of greater or less intensity, but always louder than at a later period, and diminishing during the progress of pregnancy, till it is marked by the fœtal sound. Once, when the belly was slightly pressed by the stethoscope, the murmur was heard at the upper and anterior part of the uterine tumour; and then, by continuing the compression, it ceased, giving place to the sound of the fœtal heart.

In common with Dubois, Evory Kennedy, and others, Breventani ascribes the placental sound to the uterine vessels in contact with the placenta; and he controverts the opinion of Bouillaud, that it is owing to the pressure upon the iliac arteries, by adducing a case in which he heard the placental murmur and the sound of the iliacs at the same time. The beatings of the fœtal heart are not long in making themselves heard after the uterine murmur; but they are usually detectible a little after the fourth month, although before the motions of the fœtus are perceptible. They generally diminish in frequency from the first moment they are heard to the end of pregnancy; but occasionally they become too rapid to be counted. The greatest number of double fœtal sounds heard was 149, the least 120, in the minute. The situation and extent of surface over which they are audible varies with the motions of the fœtus.

The next article is an account of two cases, with the dissections, from the pen of the same writer, in which the sounds of the heart were heard at a distance from the patients. In one of them a whizzing sound was audible at the distance of three paces, synchronous with the pulse, and proceeding from below the sternal end of the right clavicle, at which spot a purring tremor, contemporaneous with the pulse, was perceptible to the touch. The diagnosis was hypertrophy, dilatation of the aorta, and contraction of its orifice. The sound varied in intensity at different periods, and, a little before death, was only audible within a few inches of the chest. The diagnosis was confirmed by the dissection; but the valvular contraction was owing to an increased growth and development of the fibrous coat of the aorta, under the form of tubercular elevations projecting far into the interior of the vessels, obstructing its orifice, and extending in scattered clusters of less size along the descending portion. This degenerated structure contained some traces of atheromatous matter. The symptoms and morbid appearances of the second case resembled the first in every respect, but that the aortic valves were ossified, and the vessel was simply dilated.

Notwithstanding that, in almost all the dissections of such cases, the same morbid states have been found, yet, as there are numerous instances of the same lesions unattended with this peculiar loudness of sound, and that the noise itself varies in intensity, and in some cases has become inaudible except by the stethoscope, at the same time that the heart's impulse was augmented, the author is disposed to attribute it to a state of rarefaction of the blood, either by "the expansile vapours" of Rosa, or "the gases" of Testa, or "the nervous fluid" of Lobstein; as it is well known that thin fluids, when shaken, produce a far louder sound than thick ones.

Doubtless the crasis of the blood is liable to frequent variations in the same individual; and the phenomenon of the "bruit de diable" of Bouillaud and others goes far to prove that a poor and watery state of the circulating fluid is a condition favorable to the production of sound in the containing vessels; but, with respect to the three expansile or dilating matters above mentioned, we must withhold our assent to an explanation resting upon notions so purely hypothetical.

There is a very good digest of all that is known upon the subject of

Lithotrity, by Dr. Baroni; followed by a Latin dissertation by the president of the Society, Dr. Valori, upon the question whether Celsus was a practitioner as well as a writer; which question, from the internal evidence afforded by his works, is decided in the affirmative.

The last paper in the first Fasciculus is upon the Symptoms, Laws, and Peculiarities of the Cholera, by Dr. Versari; which, though a valuable compilation, contains little that is original, either in theory or practice. Dr. V. is inclined to refer the remarkable absence of tears and complaints in the patients to the general arrest of all the secretions, except those from the stomach and bowels, rather than to any peculiar state of the sensorium; though he does not altogether deny that this may have some influence. Our own experience on this point, derived from the observation of some hundreds of cases, leads us to attribute this curious state of apathy to a "lesion of innervation," produced by the retarded flow of highly carbonized blood through the brain, that deadens the perceptive faculties as well as weakens the muscular powers; besides that the mere want of the secretion of the tears is not sufficient to account for the absence of all other signs of grief and terror. In speaking of the empty state of the arteries, Versari quotes a cruel experiment of Dieffenbach's, mentioned in the *Annali* of Omodei, No. 184, in which an elastic tube was passed from the upper part of the brachial artery to the heart of a patient in his last agonies, without giving exit to a particle of blood. He considers cholera to be a peculiar disease, affecting more particularly the ganglionic system, and secondarily those organs, as the stomach, bowels, &c., with which it has the closest connexions. Like most of the Italian physicians, he adopts the opinion of its contagious nature, but not without a severe scrutiny of the arguments for and against it. Of all remedial means, he places the greatest reliance upon an early bleeding; and speaks very favorably of the use of ice, swallowed slowly and in small quantities. In the cold stage he uses stimulants, astringents, and aromatics; but with little expectation of a favorable result. The consecutive fever he treats, in the usual manner, with salines and diluents.

The first article of the second Fasciculus is an account of a Monster by Defect, by Dr. Spessa, of Crespino. This monster appears from its size, (sixteen inches long,) to have been born at the full period, and presented the following anomalies:—There was no connexion, by means of an umbilical cord, between the fœtus and the placenta, the fœtal portion of which was wanting; and, where it was attached to the uterus for a hand's breadth, it was thin and broken down. The liver and small intestines projected through a large round opening in the abdominal parietes, four inches in diameter, the edge of which was cicatrized, except for a space of three inches towards the left hypochondrium, where it was sloughy. The cord, had there been one, would have occupied the centre of the opening. An immense spina bifida occupied the whole vertebral column, from the lower part of the neck to the coccyx, containing five pounds of straw-coloured serum, and communicating with the spinal canal by numerous openings, caused by the absence of the spinous processes. The medulla, that floated in the fluid, as well as the brain, was very soft and diffluent. The feet and legs were turned inwards, so that the toes met each other. The pelvis was very narrow, and there was neither anus nor organs of generation. There was no sternum, nor cartilages to the ribs. The thymus was large; the lungs were merely rudimentary, and the pericardium and mediastinum were absent. The want of a diaphragm made but one cavity of the chest and abdomen, in which were neither spleen, omentum, nor stomach; for the œsophagus passed directly into the small intestine that terminated at the ileum in a cul-de-sac. The pancreas was small, and the kidneys had neither pelves nor ureters; nor was there any bladder or internal sexual organs; indeed, the pelvis was an irregular mass of bones, without any cavity. The liver was of an irregular form, and the gall-bladder was deeply sunk into its substance. The remains of the umbilical vessels were impervious cords. The distribution of the blood-vessels and nerves was as regular as it could be with the absence of so many important viscera.

The following is the explanation given of this extraordinary monstrosity:—1st. That there was an original defect of all the absent parts, together with the existence of an umbilical hernia, containing the liver and intestines. 2d. That this, compressed by the uterus against the brim of the pelvis, became inflamed and sloughed, forming the large opening into the abdomen, (not entirely healed at the time of birth,) besides separating the union of the fœtus and the mother; and that, 3d, The fœtus, thus separated, was enabled to maintain its independent existence nearly to the period of birth; and that this separate existence continued some time is evident from the healing of the edges of the abdominal opening, and the contracted state of the remains of the umbilical vessels. The author does not attempt to explain the way in which the nutrition of the fœtus was carried on at this period; but mentions a case from Poujol, where the umbilical arteries were both impervious. It is to be regretted that no account is given of the contents of the intestines, nor of the lymphatics and lacteals; as, by means of an examination of these organs, some light might perhaps have been thrown upon the *vexata quæstio* of the nutrition of the fœtus; particularly as it has been strongly denied by Adelon, that a fœtus has ever arrived at maturity whose umbilical cord has been divided while in utero.

In the same Fasciculus is another account, by Dr. Luigi Calori, of a Monster by Defect, in which, externally, the nose and nostrils were wanting, while a hare-lip and cleft palate converted the mouth and nasal fossæ into one cavity. To this deformity was added a vast serous cyst within the skull, compressing both hemispheres, particularly the right, of which only a rudiment remained. The corpora striata, the olfactory nerves and lobes, the commissures, the septum, fornix, and the mamillary processes, were all absent, and the carotids were much smaller than usual. There was no æthmoid bone, and the sphenoid was deficient in those parts that are connected with the nose; besides that there were many minor deformities within the skull dependent upon these more important anomalies. This "monster" lived eleven days, and then died jaundiced; but its death appeared to result from inanition, from the difficulty of taking food, rather than from any disease.

Professor Calori considers this case to be confirmatory of the law laid down by Meckel, "that, when the facial portion of the skull is deficient, the anterior part of the brain is imperfectly formed;" likewise, that the proximate cause of the deformity was (in accordance with the views of Serres,) the want of development of the carotid arteries, upon which these parts were dependent for their nutrition; the remote cause being the pressure exercised upon the parts within the cranium by the contents of the serous cyst; the formation of which he attributes to inflammation set up at an early period of the fœtal existence. With but little respect for the tender and musical ears of his countrymen, Dr. Calori has denominated this monstrosity as *Coloborrhinocephalus Fissilabrus!*

The two remaining articles are upon Scurvy, as it appeared in the prison at Narni; and on a case of Ossification of the Costal Pleura and Cartilages, attended with dyspnœa, constant, and unrelieved by any posture or mode of treatment: neither paper, however, has any claims to originality, either in its pathology or treatment; indeed, there is a great deficiency of this quality in most of the Italian periodical publications, which is hard to be accounted for, consistently with the knowledge of the works of contemporaneous authors therein displayed, unless it arise from the Italian physicians being fonder of reading than of observation.

---

